# Our Say on the Subject

**Published:** 1886-02

**Authors:** 


### OUR SAY ON THE SUBJECT.

We have endeavored to express our sentiments on all subjects of importance that come up in the medical world, but there is one which we are painfully conscious of having neglected. We feel as though our readers and the medical press of the country, if they ever thought about it, expect us to say something about "Pasteur and Inoculation for Hydrophobia." This subject has been a veritable "find" for the medical journals; it has been fruitful of "leaders" and of "minor paragraphs;" but up to date, the "voice of Texas" has been silent on the mighty theme.

Now, if a man have a rent in his trousers, or a hole in his shoe, he is painfully aware of it; his mind dwells on it; he is unconfortable; and when he goes out, he is quite sure that every body sees it, knows it, and is commenting unkindly on it. It is said that Byron's consciousness of a deformed foot embittered his whole life; it was a most painful subject to him. We do not mean to say the foot was painful, but a knowledge that it was not like other people's feet caused him pain. He allowed it to outweight—counterbalance all the glory which was heaped upon him, all the praise, admiration and envy which the world showered on his brilliant genius and productions. This phase in human nature has always puzzled us.

But, to make the application: We are painfully aware of a neglect of duty on our part,—the failure to write a "leader" on Pasteur and his "Inoculations," and we feel that everybody has observed it. This is the hole in our coat, our deformed foot. Well, we have seriously contemplated it. We have been like the boy who is being coaxed,and driven,and bribed to take the oil. We have tried to make up our minds to attack the subject, and have squared off at it several times; but so far, we havn't dared to tackle it; feeling sure we *can't* swallow it. This inoculation-with-the-marrow-of-a-dead-rabbit's - back - bone-business is too much for our digestion. Yet all the other journals have had a whack at it; all have popped their guns at it; broadsides have been fired into it, (but at last accounts Pasteur was inoculating still,) and here *we* are, feeling that our readers are saying, "why don't *you* blaze away?" and are surprised and indignant at our silence, and, with frowning brows, and clenched fists, are severely holding as responsible. We feel like a culprit, a wretched sinner.

But what shall we do? Shall we endeavor, at this late day, to get off something learned about it? out with an "opinion" any how? That would be folly; all the good "opinions" have been expressed; all the stereotyped phrases, either in condemnation or approval, have been utilized, woven into editorials of more or less wind or wisdom, and there is nothing left, in the way of stock, on which to construct an editorial! Shall we apologize? Worse than ever; for, perhaps, after all, may be, most likely, nobody has noticed the omission on our part, and it is only our vanity that makes us think they have—but, all same, we feel like there is a rent in the rear of our journalistic trousers, that our tripod-ic foot is crooked (ahem!), and we are uncomfortable, and *must* say something to relieve our mind (or our foot? or the hole in our pants?) The fact is, we *haven't* any opinion on the subject; it is past our comprehension; but here goes:

In the first place, according to all rule, precedent and former observation, the introduction of dead animal matter into the circulation of a living human being *ought* to produce blood poisoning, to say nothing of local trouble; and, as the introduction of a particle of this same back-bone marrow into the economy of a cat or rabbit seems to kill that creature pretty quick, *why* does it not kill the man or boy? Is it because the man or boy has been bitten by a mad-dog and the cat hasn't? And does the poison of the mad-dog neutralize that of the spinal marrow? Suppose it should happen that the dog wasn't mad? Eh!

Seriously, the process seems opposed to all reason, experience and common sense; and had any other than Pasteur or Koch proposed it—from whose lips the world has learned to look for words of wisdom only, had an *unknown* seriously tried to prevent the development of rabies by inserting under the skin of a man or boy a portion of the spinal substance of any dead animal, much less that of one which had died violently of hydrophobia, he would have been thought crazy! Has *Ferran* been so soon forgotten?

American physicians are by Pasteur like Catherine was by Petrucio; when Petrucio declared "'tis the moon" which shone so brightly at noon, Kate swore to it.

We are a set of flunkies!

